# Repeatability of Ad Libitum Water Intake during Repeated 1 h Walking/Jogging Exercise Sessions Conducted under Hot Ambient Conditions

**DOI:** 10.3390/nu15214500

**Published:** 2023-10-24

**Authors:** Eric D. B. Goulet, Pascale Claveau, Ivan L. Simoneau, Thomas A. Deshayes, Antoine Jolicoeur-Desroches, Fedi Aloui, Martin D. Hoffman

**Affiliations:** 1 Faculty of Physical Activity Sciences, University of Sherbrooke, Sherbrooke, PQ J1K 2R1, Canada; pascale.claveau2@usherbrooke.ca (P.C.); thomas.deshayes@usherbrooke.ca (T.A.D.); antoine.jolicoeur-desroches@usherbrooke.ca (A.J.-D.); fedi.aloui@usherbrooke.ca (F.A.); 2 Research Center on Aging, University of Sherbrooke, Sherbrooke, PQ J1H 4C4, Canada; 3 Centre de Recherche et de Formation par Simulation, Cégep of Sherbrooke, Sherbrooke, PQ J1E 4K1, Canada; drsimoneau1@gmail.com; 4 Independent Consultant, El Dorado Hills, CA 95762, USA; md.hoffmanmd@gmail.com

**Keywords:** dehydration, drinking pattern, exercise, heat stress, physiological and perceptual functions

## Abstract

A drinking strategy aiming to replace a given percentage of the sweat losses incurred during exercise should result in reproducible fluid intake volume and, hence, fluid balance from one exercise session to the other performed under similar scenarios. Whether this may also be the case with ad libitum drinking during exercise is unclear. We characterized the repeatability of ad libitum water intake during repeated 1 h exercise sessions and examined its effect over time on fluid balance and selected physiological functions and perceptual sensations. Twelve (3 women) healthy individuals participated in this study. At weekly intervals, they completed four 2 × 30 min walking/jogging exercise bouts (55% $\dot{V}$O_2max_, 40 °C, 20–30% relative humidity) interspersed by a 3 min recovery period. During exercise, participants consumed water (20 °C) ad libitum. There were no significant differences among the four exercise sessions for absolute water intake volume (~1000 mL·h^−1^), percent body mass loss (~0.4%), sweat rate (~1300 mL·h^−1^) and percent of sweat loss replaced by water intake (~80%). Heart rate, rectal temperature, and perceived thirst and heat stress did not differ significantly between the first and fourth exercise sessions. Perceived exertion was significantly lower during the fourth vs. the first exercise session, but the difference was trivial (<1 arbitrary unit). In conclusion, ad libitum water intake during four successive identical 1 h walking/jogging sessions conducted in the heat will result in similar water intake volumes and perturbations in fluid balance, heart rate, rectal temperature, and perceived thirst, heat stress and exertion.

## 1. Introduction

Humans produce sweat during exercise which, if properly evaporated, limits the increase in core body temperature [1]. Hence, the consumption of fluid during exercise is important to maintain the body’s water levels within an acceptable spectrum. Indeed, improper consumption of fluid during exercise may lead to excessive dehydration, which may increase plasma osmolality, glycogen usage and perceived exertion and thirst and reduce mood, plasma volume, thermoregulatory ability, cardiovascular efficiency, and endurance performance [2,3,4]. 

Before exercising, individuals must determine whether fluid replacement is required. If the answer is positive, the person then needs to decide on how fluid should be replaced during that exercise. There are two main hydration strategies that individuals may privilege during exercise: (1) drink in a planned manner or (2) drink in an ad libitum manner [5]. The decision to choose one strategy over the other should be based on the exercise circumstances, fluid availability, gastrointestinal tolerance, fluid carrying capacity and personal preferences [6]. Independent of the chosen option and despite ad libitum drinking usually being associated with a lesser volume of fluid consumed during exercise compared with planned drinking, it is expected that both hydration strategies will have a similar effect on endurance performance over 1–2 h of running or cycling exercises [7].

When relying on planned drinking, individuals must decide beforehand on an ideal rate of fluid replacement for the upcoming exercise. According to the National Athletic Trainers’ Association (NATA) [5] and the American College of Sports Medicine (ACSM) [8], the amount of fluid replacement should be sufficient to limit the loss of body mass through sweat, urine and breathing to 2%; otherwise, endurance performance will be impacted negatively [2]. Under this circumstance, the estimated amount of fluid to be consumed for a particular exercise session should be scenario specific and established a priori through a sweat rate test [5,8,9,10]. Therefore, for exercise sessions performed under similar scenarios of exercise intensity, exercise duration, ambient temperature, absolute humidity, baseline state of hydration, ingested fluid temperature and an individual state of heat acclimatization, the extent of fluid replacement during exercise and, consequently, fluid balance during and following exercise, should be similar between exercise sessions and, hence, also the resulting hydration-driven perturbations in physiological responses to exercise. Understanding or predicting how hydration impacts the physiological responses to exercise may be important for athletes, recreationally active individuals, and coaches. Indeed, it may help them (1) improve the planning of exercise sessions, (2) increase the understanding of the level of stress induced by the exercise sessions, (3) enhance the management of the recovery periods following exercise and (4) better predict the adaptations to different exercise stimuli applied over time. 

Individuals relying on an ad libitum drinking strategy to replace fluid during exercise may start an exercise with no pre-established drinking plan, having little idea of their sweat rates and associated fluid requirements, and should consume fluids during exercise whenever they want and in whatever volume they want [11,12]. Therefore, as fluid replacement with this drinking strategy is not systematically controlled, it could potentially be more difficult for individuals to understand and predict what the physiological responses to exercise-induced hydration perturbations might be from one exercise session to another performed under similar scenarios. And, ultimately, this may increase the difficulty of predicting and understanding the responses to exercise and planning optimal recovery strategies.

Although the term ad libitum drinking theoretically refers to a nonstructured behavior, Claveau et al. [11] demonstrated that requiring athletes to drink either ad libitum or based on their thirst perception leads to identical volumes of fluid consumed during prolonged cycling exercise. Therefore, ad libitum driven fluid intake during exercise appears to be dependent, at least in part, upon the perception of thirst. To this effect, Maresh et al. [13] have shown that hypohydration preceding exercise in the heat modulates thirst-driven fluid intake during exercise and results in similar fluid regulatory hormonal responses and alterations in plasma volume, compared with euhydration. Moreover, studies in rodents have demonstrated that increases in core body temperature induce significant fluid intake prior to any alterations in extracellular fluid osmolality or volume occur [14]. Hence, hyperthermia can initiate fluid ingestion in anticipation of the sweat losses that would take place in the future to mitigate the increase in core body temperature. Altogether, the above observations suggest that ad libitum driven fluid intake may be involuntarily regulated by individuals and, hence, could potentially result in predictable fluid intakes during exercise conducted under similar scenarios. 

We found less than a handful of relevant studies [15,16,17,18] that examined whether ad libitum fluid intake may result in reproducible fluid intake volumes under identical exercise scenarios. It must be noted that this literature is not readily identifiable, as the characterization of ad libitum fluid consumption during exercise as the main study’s goal is not explicitly appreciable from the available titles. Altogether, the results of these studies are mixed and produce more questions than answers. Wilk et al. [17] had 10 to 12 years old boys perform 6 exercise sessions over a 2-week period. A sports drink (grape-flavored, 6% carbohydrate) was consumed ad libitum during exercise. Fluid intake was reproducible from one session to the other, but it remains unknown whether the results translate to men. Another study [15] examined the ad libitum fluid intake of women over three distinct phases of the menstrual cycle. Women performed three intermittent exercise sessions (30 min exercise/30 min rest) in the heat over a period of several weeks. Ad libitum fluid intake (flavor-preferred, noncaloric sports drink (Gatorade Zero^TM^)) was only permitted during the breaks. Again, fluid intake was repeatable, but it is unclear what results would have been observed had participants been permitted to drink only during exercise, not only during the breaks. Greenleaf et al. [18] asked men to complete 2 h of exercise (23.8 °C, 50% relative humidity) per day for 8 consecutive days while consuming tap water (16 °C) in an ad libitum manner. The fluid intake did not vary significantly between exercise bouts. It is unclear if those results would apply under warm/hot conditions where sweat production is greater and thirst more challenged than in a thermoneutral climate. Finally, Sekiguchi et al. [16] showed that ad libitum fluid intake (water) did not result in similar fluid intake volumes in participants performing five consecutive days of heat acclimation training. Whether these results would hold during nonheat acclimation training remains to be determined. 

There is a need to further our knowledge and observe and characterize the repeatability of the ad libitum water intakes of healthy adults over several exercise sessions performed under identical exercise scenarios. Therefore, the first objective of this study was to determine whether the ad libitum associated variations in the amount of water consumed, percentage of sweat loss replaced, and dehydration level differ significantly between identical 1 h exercise sessions repeated on multiple occasions over time. The second objective of the study was to determine whether parameters that can be impacted by water intake during exercise, such as heart rate, rectal temperature and perceived thirst, heat stress and exertion, differed across exercise sessions. We hypothesized that the volumes of water consumed through ad libitum drinking would not differ significantly from one exercise session to the other and, therefore, that the evaluated physiological functions and perceptual sensations would also not be significantly altered. 

## 2. Materials and Methods

### 2.1. Participants

Twelve (3 women) healthy, physically active and nonheat-acclimatized individuals participated in this research project. Heat acclimatization induces several adaptations that improve the control of fluid balance [19]. The potential confounding impact of this factor on the findings was therefore controlled for, with recruitment being limited to nonheat-acclimatized participants instead of a combination of heat- and nonheat-acclimatized individuals. Their physical characteristics are presented in Table 1. Prior to the initiation of any procedures, the nature, risks and associated benefits of the study were fully explained to the participants. Then, they signed an informed consent form. The research protocol and informed consent form were approved by the CIUSSS Estrie-CHUS Ethics Committee (project #2019-3081). 

### 2.2. Context of the Study

**Figure 1:**
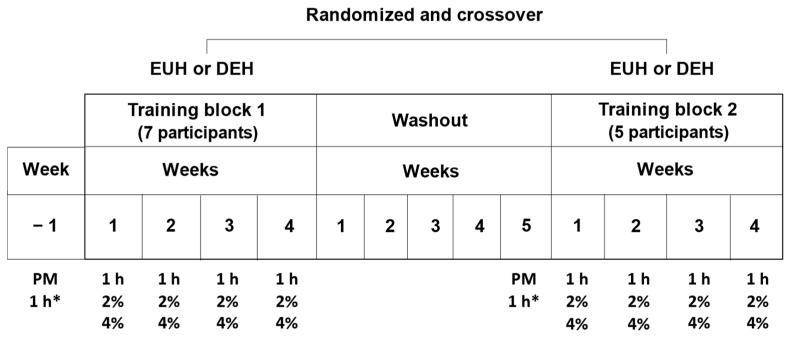
Schematic of the mother research protocol, illustrating the distribution of the 1 h exercise sessions during the training blocks. PM: preliminary measurements; 1 h*: 1 h exercise session in which water intake was not monitored and individualized exercise intensity determined and confirmed; 1 h: 1 h exercise session in which ad libitum water intake was monitored; 2%: exercise until a 2% body mass loss has been lost or replaced by water intake; 4%: exercise until a 4% body mass loss has been lost or replaced by water intake; EUH: euhydration; DEH: dehydration.

This study was an integral part of a larger research project whose goal was to examine the impact of habituation to exercise-induced dehydration on endurance performance and cognition. The results of these studies have already been published [20,21]. As illustrated in Figure 1, these studies required participants to complete two 4-week training blocks (walking/jogging on a treadmill) in the heat interspersed by a 5-week washout period. The goal of the washout period was to eliminate the heat stress- and hypohydration-related adaptations that may have taken place during the first training block. The training blocks were administered in a randomized and crossover fashion. Each training block was composed of 3 exercise sessions per week, each spaced by 48–72 h. The first training session of the week always consisted in a 1 h exercise session where participants were required to drink ad libitum. For the remaining 2 exercise sessions, during one they were required to exercise until a 2%, and in the other, until a 4% body mass has been lost (dehydration training block) or all fluid loss was replaced (i.e., euhydration training block). Only the results of the 1 h exercise sessions completed during the training block in which the participants were required to remain well hydrated (euhydration training block) during the 2% or 4% body mass loss exercise sessions are presented in this study. Seven participants completed the 1 h exercise sessions during their first training block and the remaining five during their second training block after the washout period had been completed. Therefore, our results are representative of individuals who participated in a total of four 1 h exercise bouts interspersed by 7 days. However, 1-week prior to starting each training block, the participants came to the laboratory for the (1) collection of preliminary measurements and (2) completion of a 1 h exercise session to familiarize themselves with the environmental conditions and determine and confirm the proper exercise intensity to be used in all upcoming exercise sessions. Subjects could drink ad libitum during this specific session, but the water volume consumed was not measured as the exercise intensity was not fixed throughout the exercise period. 

### 2.3. Preliminary Measurements

Body mass while postvoid was measured with an electronic floor scale (±20 g, BX-300+, Atron Systems, West Caldwell, NJ, USA), height using a stadiometer, and resting blood pressure and heart rate after a 3 min rest period with a digital sphygmomanometer (Welch Allyn 420 series, Skaneateles Falls, NY, USA). Fat-free mass (FFM) was assessed with dual-energy X-ray absorptiometry technology (Lunar Prodigy, GE Healthcare, Chicago, IL, USA). Maximal oxygen consumption ($\dot{V}$O_2max_) was measured on a motorized treadmill (TMX428 Trackmaster, Newton, KS, USA) using an expired gas analysis system (Cosmed Quark CPET, Cosmed, Chicago, IL, USA) that had been calibrated with gases of a known concentration. The test started at a speed of 7 km·h^−1^, with a fixed grade of 1%. Each subsequent min, the speed was increased by 1 km·h^−1^ until participants reached exhaustion. The attainment of $\dot{V}$O_2max_ was confirmed using recognized criteria [22].

### 2.4. Pre-Exercise Protocol

Participants completed the 1 h exercise sessions at a time of their choice and were required to drink 250 mL of water 60 min prior to bedtime the night before each exercise session as well as 60 min prior to their arrival at the laboratory for the exercise sessions. Participants abstained from food and fluid intake during the last 60 min prior to the exercise sessions and were requested not to exercise for the last 12 h prior to reporting to the laboratory. Otherwise, nutrition, hydration and training were not further standardized between and before exercise sessions. All exercise sessions occurred in Sherbrooke, Canada, during the spring and early summer months. 

### 2.5. 1 h Exercise Sessions

Immediately following their arrival at the laboratory, participants emptied their bladder, collected a urine sample, were weighted in the nude (±50 g, MyWeight HD-300, HBI Technologies, Phoenix, AZ, USA), dressed themselves, installed a chest electrode and inserted a rectal telemetric pill. Following these procedures, participants entered the environmental chamber (40 °C, relative humidity: 20–30%, wind speed: ~5 km·h^−1^), rested on the treadmill for 2 min and then started walking or jogging at an intensity of 55% $\dot{V}$O_2max_. The ambient temperature, relative humidity, wind speed and exercise intensity replicated the conditions encountered by participants during the 2% and 4% body mass loss exercise sessions of the mother research protocol illustrated in Figure 1. The exercise sessions consisted of two 30 min exercise bouts interspersed by a recovery period of 3 min. During exercise, participants were allowed to replace fluid with water consumed ad libitum, which was provided at 20 °C in opaque cycling bottles. Participants were not made aware that their intake of water was being monitored during exercise. Following exercise participants voided their bladder, toweled dry and were weighted. 

### 2.6. Measurements

Urine was collected using graduated urinals. Urine was weighted considering 1 g = 1 mL [23]. The urine specific gravity was assessed in duplicate using a digital refractometer (PAL-10S, Atago, Bellevue, WA, USA). Heart rate (USB ANT stick (Garmin, Olathe, KS, USA) + Golden Cheetah software) was measured continuously using a Garmin Premium chest electrode (Garmin, Olathe, KS, USA). Rectal temperature was measured every 10 min using a calibrated telemetric pill (CorTemp^TM^, HQ Inc., Palmetto, FL, USA) inserted just passed the anal sphincter [24]. Each pill was reused for 50 h [24]. A proper sterilization was performed between each use [24]. Perceived exertion (15-point Borg scale [25]), perceived thirst (11-point scale [24]) and perceived heat stress (7-point scale [24]) were measured every 10 min. Percent dehydration was computed as the difference between pre- and post-exercise body mass relative to pre-exercise body mass. Sweat loss was calculated by subtracting the post- from the pre-exercise body mass, correcting for water intake and urine losses [26]. The sweat rate (mL·h^−1^) was considered to represent the amount of sweat produced during the entire hour of exercise. Respiratory water losses and losses of mass associated with the respiratory exchange of O_2_ and CO_2_ were not considered and assumed to be similar among trials [27]. The percent of sweat loss replaced by ad libitum water intake was taken as the total volume of water consumed during exercise relative to sweat loss. 

### 2.7. Statistical Analyses

The normality of distribution of residuals (or differences for the dependent *t*-test) was tested using the Shapiro–Wilk test. One-way repeated measures ANOVAs were used to analyze pre-exercise body mass and percentage of body mass change among exercise sessions 1 vs. 2, vs. 3 and vs. 4, and overall water intake volume, sweat rate, percentage of body mass loss, percentage of sweat loss replaced, balance between water intake and sweat loss and change in mean heart rate among exercise sessions. A Friedman test was used to analyze changes in pre-exercise perceived thirst among exercise sessions. A dependent *t*-test was used to compare the change in heart rate from min 0 to 60 of the first exercise session vs. that of the fourth exercise session. Two-way repeated measures ANOVA were conducted to examine the influence of time, exercise session and their interaction on heart rate and perceived heat stress. In replacement of the one-way repeated measures ANOVAs, two-way repeated measures ANOVAs and the dependent *t*-test, linear mixed models were used in the case of missing data (pre-exercise urine specific gravity (2 (missing data)/48 (total data)), laboratory temperature (2/48) and relative humidity (2/48), change in rectal temperature over time among exercise sessions (35/336), change in mean rectal temperature among the exercise sessions (4/48) and change in rectal temperature between min 0 and 60 of the first exercise session vs. that of the fourth exercise session (3/24) and, because of its robustness to violations of distribution assumptions [28], when residuals were abnormally distributed (change in perceived exertion and thirst over time among exercise sessions). When needed, post hoc comparisons were performed using the false discovery rate procedure. Repeated measures correlations were performed to examine relationships among variables [29]. Coefficients of variation (CV) were computed using the root mean squared method [30]. Unless otherwise noted, the results are reported as the mean ± standard deviation (SD). Statistical significance was accepted as *p* ≤ 0.05. Statistical analyses were performed with the Microsoft Excel (version 2212, Microsoft, Redmond, WA, USA), IBM SPSS statistics (version 21, New York, NY, USA), MedCalc (version 22.006, Ostend, Belgium) and R studio (version 2021.09.01, Boston, MA, USA) software. 

We estimated the sample size required for this study based on the results of a previously published study [27] by our laboratory, where the repeatability of ad libitum fluid intake volume from a familiarization session to an experimental trial during 1 h of cycling was computed (change in fluid intake volume = 101 mL; effect size = 0.66, *r* = 0.104). From these numbers, it was determined that 7 participants were required to detect a statistically significant effect (1 – β = 0.8, α = 0.05) in fluid intake volume between exercise sessions using a one-way repeated measures ANOVA with one group of subjects and 4 measurement periods. The G*Power software was used to compute sample size (version 3.1.9.6., Kiel University, Kiel, Germany). 

## 3. Results

### 3.1. Pre-Exercise Hydration and Ambient Temperature and Relative Humidity

Table 2 presents values of urine specific gravity, body mass and perceived thirst observed before each exercise session as well as the percentage change in pre-exercise body mass from exercise session 1 to exercise sessions 2, 3 and 4. Despite statistically significant changes in the urine specific gravity between exercise sessions and the percentage change in body mass between exercise sessions 1 vs. 2, 3 and 4, altogether, these results suggest that participants were well and similarly hydrated prior to each exercise session. The mean laboratory ambient temperature (39.8 ± 0.5 °C, *p* = 0.28) and relative humidity (24.7 ± 2.4%, *p* = 0.16) did not differ among exercise sessions.

### 3.2. Fluid Balance

**Figure 2:**
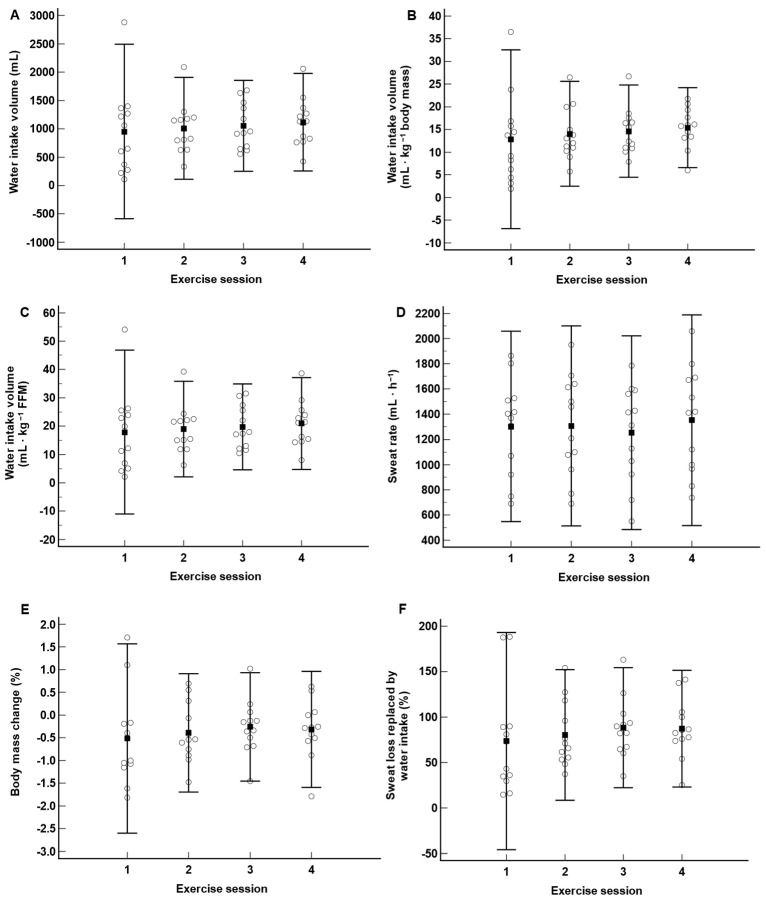
Distribution of absolute water intake volume (**A**), water intake volume corrected for body mass (**B**), water intake volume corrected for fat-free mass (**C**), sweat rate (**D**), body mass change (**E**) and percentage of sweat loss replaced by water intake (**F**) over the four exercise sessions. Unfilled circles: individual responses; filled squares: mean responses; bars: 95% confidence intervals.

Figure 2 depicts the individual and mean, as well as the distribution (95% confidence interval (CI)), of the absolute and relative (per body mass and FFM) water intake volume, percent body mass loss, sweat rate and percent of sweat loss replaced by water intake during each exercise session. There were no statistically significant differences (all *p* > 0.36) among the four exercise sessions for any of the variables. Over the four exercise sessions, the mean sweat rates (~1300 ± 384 mL·h^−1^) were slightly higher than the mean absolute water intake volumes (~1000 ± 520 mL·h^−1^), which led to mean body mass changes of ~−0.4 ± 0.7%. Hence, participants replaced a significant portion (~80 ± 41%) of their sweat losses through water intake during exercise. The variations in the mean absolute water intake volume between exercise sessions 1 and 2, 2 and 3 and 3 and 4 were 58 ± 351, 43 ± 342 and 64 ± 293 mL. The most important variation occurred between exercise sessions 2 and 4, with 164 ± 623 mL. Table 3 shows the balance between the total accumulated water intake volume and total accumulated sweat loss for each of the exercise sessions and over the four exercise sessions. For each of the four exercise sessions, the mean absolute water intake volume was lower than the mean sweat loss, with no significant difference between exercise sessions (*p* = 0.56). Five individuals, at least for one exercise session, consumed more water than they lost through sweat. Three individuals completed the four exercise sessions with a positive fluid balance. 

Within- and between-exercise-session variations in absolute water intake volume were important. Indeed, the between-subjects CVs for absolute water intake volume during exercise sessions 1, 2, 3 and 4 were, respectively, 80.9, 44.5, 38.2 and 38.6%. On the other hand, the within-subjects CVs for absolute water intake volume between exercise sessions 1 and 2, 2 and 3 and 3 and 4 were, respectively, 38.1, 22.4 and 18.4%. Hence, the within-subjects CVs were approximately two-fold lower than the between-subjects CVs. Correcting for either body mass or FFM did not substantially change these outcomes. Perceived thirst did not explain the variations in water intake volume between nor within individuals. Indeed, there were no correlations between absolute water intake volumes and mean perceptions of thirst (r = 0.09, *p* = 0.58) or changes in absolute water intake volume and the changes in perception of thirst (r = −0.12, *p* = 0.57) from one exercise session to the other. Also, no significant correlation (r = 0.02, *p* = 0.90) was observed between absolute water intake volumes and sweat rates over the four exercise sessions. However, a significant (r = −0.60, *p* < 0.01) negative relationship was observed between absolute water intake volumes measured during exercise sessions 1, 2 and 3 and the percentage changes in absolute water intake volume between exercise sessions 1 and 2, 2 and 3 and 3 and 4. Finally, we observed no significant relationship (r = −0.01, *p* = 0.96) between sweat rates and perceived thirsts. 

### 3.3. Rectal Temperature and Heart Rate

**Figure 3:**
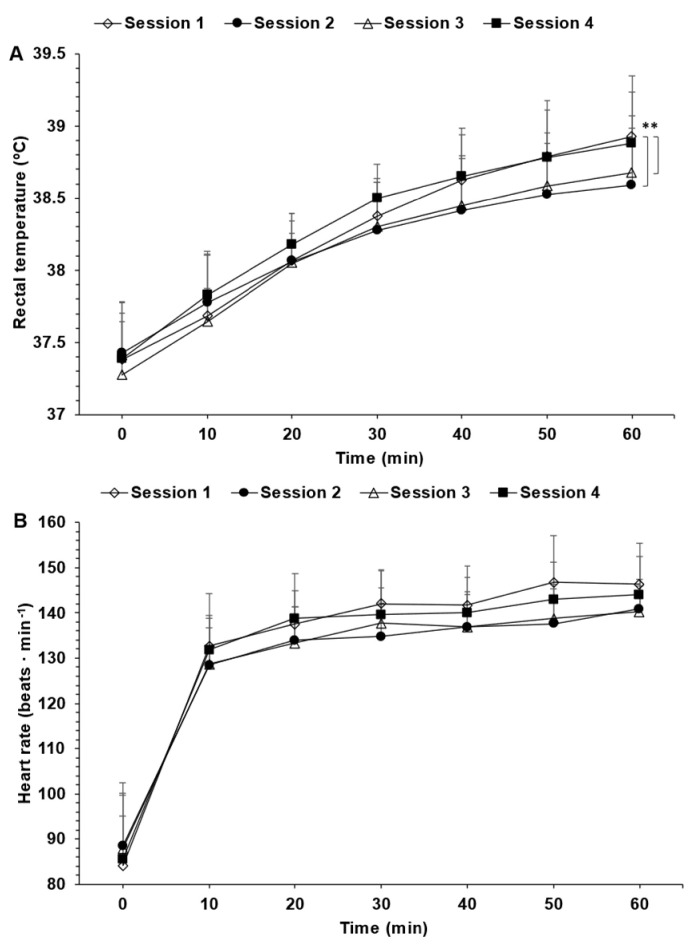
Change in rectal temperature (**A**) and heart rate (**B**) observed during each of the four exercise sessions. * *p* < 0.05. Data are the means ± SD.

Rectal temperature (Figure 3A), as well as heart rate (Figure 3B), increased continuously over time during each of the exercise sessions. A time (*p* < 0.01), but no exercise session (*p* = 0.06) or interaction (*p* = 0.06) effect, was detected for heart rate, whereas for rectal temperature, exercise session (*p* = 0.02) and time (*p* < 0.01) but no interaction (*p* = 0.54) effects were observed. Mean changes in heart rate from exercise sessions 1 vs. 2, 1 vs. 3 and 1 vs. 4 became progressively lower over time, but post hoc analyses revealed no significant differences among exercise sessions. There was no significant change between the change in heart rate (min 60 − min 0) of the first exercise session vs. that of the fourth exercise session (*p* = 0.30). The same pattern of change as that observed with the mean heart rate occurred for mean rectal temperature from exercise sessions 1 vs. 2, 1 vs. 3 and 1 vs. 4, with the exception that post hoc corrections revealed significant differences for exercise sessions 1 vs. 2 and 1 vs. 3, but not for exercise session 1 vs. 4. It must be noted that none of the differences between exercise sessions exceeded 0.15 °C. There was no significant change between the change in rectal temperature (min 60 − min 0) of the first exercise vs. that of the fourth exercise session (*p* = 0.57). 

### 3.4. Perceived Thirst, Exertion and Heat Stress

**Figure 4:**
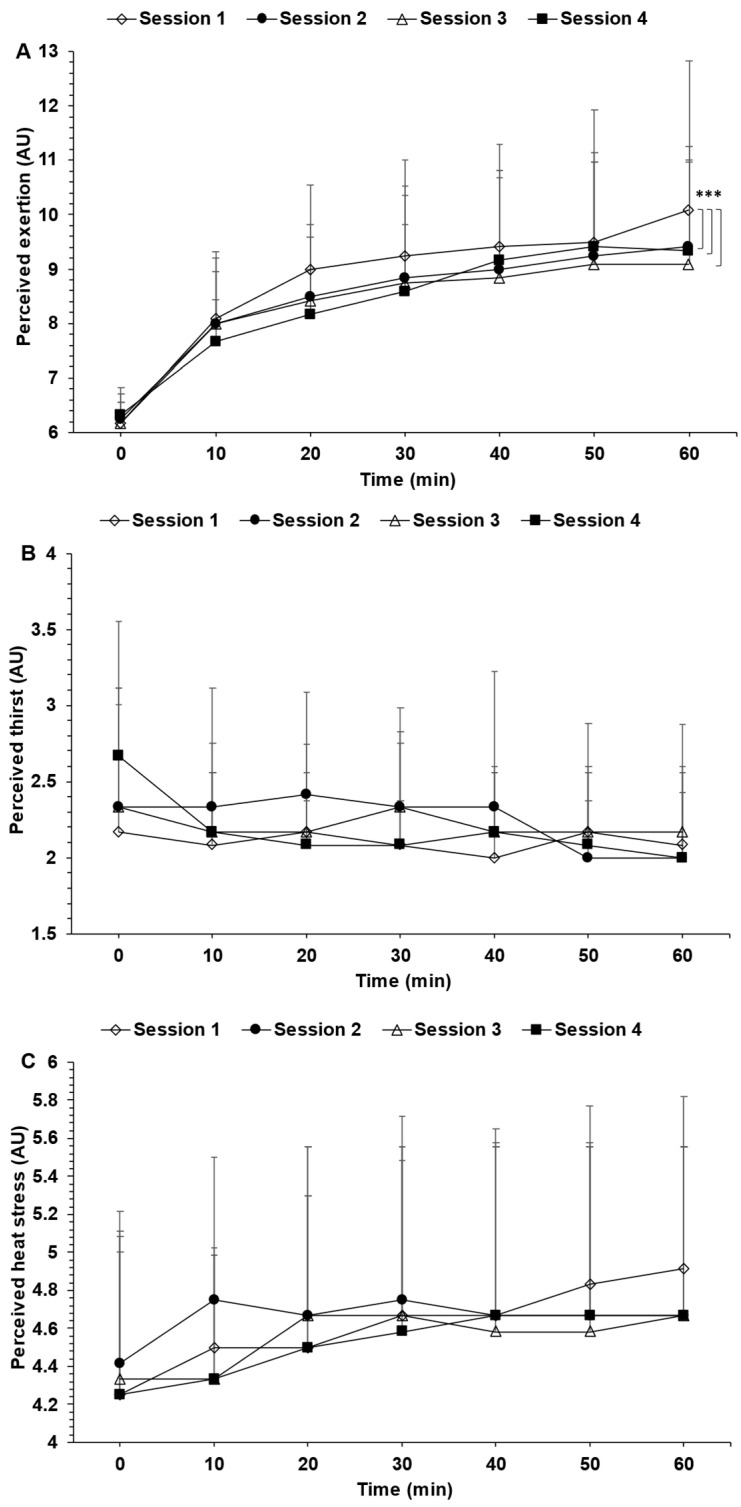
Change in perceived exertion (**A**), perceived thirst (**B**) and perceived heat stress (**C**) observed during each of the four exercise sessions. AU: arbitrary unit. * *p* < 0.05. Data are the means ± SD.

Perceived thirst (Figure 4B), as well as perceived heat stress (Figure 4C), were not associated with significant time, exercise session or interaction effects. However, exercise session (*p* = 0.01) and time (*p* < 0.01) but no interaction (*p* = 0.96) effects were detected for perceived exertion (Figure 4A). Post hoc corrections revealed that the changes in perceived exertion for exercise sessions 1 vs. 2, 1 vs. 3 and 1 vs. 4 were all significant, although they could be considered trivial as they were all less than 1 AU.

## 4. Discussion

In this study, we characterized the repeatability of ad libitum water intake by healthy adults over four identical 1 h exercise sessions interspersed by 7 days and determined how this drinking strategy would impact fluid balance, heart rate, rectal temperature, perceived exertion, perceived thirst and perceived heat stress over the four exercise sessions. To the best of our knowledge, this is the first study to shed a direct spotlight on the drinking behavior of adults required to consume water in an ad libitum manner over multiple exercise sessions completed under uniform exercise conditions (40 °C, relative humidity: 20–30%, walking/jogging at 55% $\dot{V}$O_2max_).

This study was part of a larger research project in which participants were required to perform two additional weekly exercise sessions under hot conditions where they replaced fluid losses with equal amounts of water. Yet, no signs of heat acclimation were observed in our participants over the four exercise sessions, as suggested by the lack of significant difference in mean heart rate, rectal temperature and sweat rate between the first and last exercise sessions [31]. Therefore, within the context of the current study in which participants were unacclimated to the heat, our findings show that consuming water ad libitum results in similar fluid intake volumes, heart rates, rectal temperatures and perceptual sensations from one exercise session to the other realized under identical circumstances. In our opinion, the results of this study are relevant and important. Indeed, they could help athletes, recreationally active individuals, and coaches understand how an ad libitum drinking strategy influences the volume of fluid consumed under similar exercise conditions and, as a result, improve the (1) anticipation of possible dehydration-driven physiological and perceptual responses to exercise; (2) planning of exercise sessions; and (3) management of the recovery periods.

Ad libitum drinking resulted in reproducible water intake volumes over the four exercise sessions. The greatest difference in the mean water intake volume observed among any of the exercise sessions was 165 mL, which occurred between the first and fourth exercise session. Put into perspective, this amount is relatively trivial, corresponding to less than one-quarter to one-third of a regular 600–750 mL cycling water bottle or, at most, to approximately three sips of fluid for healthy young adults, at least during cycling exercise [11]. 

This finding agrees with that found in the study of Wilk et al. [17], who wanted to determine whether the ad libitum consumption of sports drink over six identical cycling sessions of 70 min performed in the heat could systematically protect young boys from dehydration. Indeed, they observed a nonsignificant maximal variation in fluid intake volume of 137 mL among the exercise sessions. Our finding is also in line with that of Freemas et al. [15], who wished to determine the role played by the different menstrual cycle phases (i.e., follicular, late follicular and mid-luteal phases) on ad libitum fluid intake volume over three identical intermittent exercise sessions conducted in the heat. They reported a nonsignificant maximal variation in fluid intake of 304 mL among the exercise sessions. Greenleaf et al. [18] also arrived at similar results. They required five men to complete 2 h of exercise per day in a thermoneutral climate for 8 consecutive days while consuming tap water ad libitum. Fluid intake did not vary by more than 103 mL among exercise sessions. Sekiguchi et al. [16] examined the ad libitum fluid intake volume of endurance athletes undergoing five consecutive days of heat acclimation training. Contrary to our results and those of Wilk et al. [17], Freemas et al. [15] and Greenleaf et al. [18], they observed a significant difference among the exercise sessions with a maximal variation in fluid intake of 540 mL observed between training sessions 1 and 5. 

It is unclear how the results of these studies can be compared to ours, as the first provided sports drink and was not performed in adults but rather in young boys, the second had participants perform their exercise sessions at several weeks of interval and allowed them to consume fluids only during the resting periods, the third was completed in a thermoneutral climate and, for the fourth, the exercise intensity was not fixed and the exercise sessions occurred during a heat acclimation process. But a tentative synthesis of the available literature could be that for young- to middle-aged males and females, ad libitum fluid consumption under identical exercise conditions completed in thermoneutral and hot conditions could lead to reproducible fluid intake volumes, unless consecutive exercise sessions occur in the heat in nonheat-acclimatized individuals. This topic needs to be researched further before more solid conclusions can be drawn.

Whether examined in terms of absolute change or corrected for body mass or FFM, our findings demonstrate that there was substantial variation in ad libitum water intake volume among participants. Indeed, the CV for absolute water intake volume among participants was of the order of 80% for the first exercise session, but this figure reduced drastically to ~40% for the remaining three exercise sessions. We interpret this finding to suggest that participants likely learned from the experience gained during the first exercise session and adjusted their water intake volume towards a more physiologically common and narrower spectrum of water intake over the remaining exercise sessions which, according to Figure 1A, was between 500 and 1500 mL. The within-participants’ CVs for water intake volume between exercise sessions 1–2, 2–3, and 3–4 support the previous assertion and add the notion that participants continued to adjust their ad libitum water intake volume from exercise sessions 2 to 4 at a relatively constant rate of ~±200–400 mL. This point is reinforced by the fact that we observed a significant relationship between absolute water intake volumes measured during exercise sessions 1, 2 and 3, and the percentage changes in absolute water intake volume between exercise sessions 1 and 2, 2 and 3, and 3 and 4, respectively. Hence, individual water intake volumes from exercise sessions 1, 2 and 3 seem to have influenced the changes in water intake volume during each of the following exercise session. Altogether, individuals should expect that variations in ad libitum fluid intake volume of less than 500 mL should occur from one exercise session to the other conducted under similar conditions. 

It is unclear what drove the inter- and intra-individual differences in water intake. Differences in sweat rates among participants, impacting the rate of water loss and change in plasma osmolality, both modulators of thirst [32], could be a possible answer, but we observed no relationship between sweat rates and water intake volumes or perceived thirst, and there was no divergence in sweat rates among exercise sessions. Additionally, we observed no association between the ratings of perceived thirst and water intake volumes within exercise sessions nor between the changes in the ratings of perceived thirst and water intake volumes among exercise sessions. For each of the exercise sessions the ratings of perceived thirst were kept low and varied insignificantly over time. Therefore, it is not impossible that participants consumed water ahead of the development of their thirst or that our timing of the perceived thirst assessment during the exercise sessions with respect to that of the participants was not optimal, both of which would have led to the impossibility of detecting a relationship between thirst and water intake volume or sweat rate. 

According to the ACSM [8], the goal of drinking during exercise is to prevent a body mass loss >2%. The results in Figure 2E show that the participants’ rate of sweat loss replacement through water intake was sufficient to maintain the change in body mass below this threshold at the end of each exercise sessions. Hence, under the current exercise scenario, it seems that ad libitum drinking offered acceptable protection against deleterious decreases in body mass during exercise. However, one must recognize that under this particular exercise scenario, only a few individuals were susceptible to losing more than 2% body mass, even if water consumption had been withheld. More specifically, based on total sweat loss and initial body mass, only four different individuals over the four exercise sessions could have lost a body mass >2% with water deprivation.

On the other hand, ad libitum drinking did not protect some of the participants from overdrinking during exercise. In fact, an inspection of the findings in Table 3, as well as in Figure 2E,F, indicates that during each of the exercise sessions, two to three participants consumed more water than they lost through sweat. Moreover, three individuals completed the four exercise sessions with a positive fluid balance, i.e., they had consumed more water over the four exercise sessions than they had lost through sweat. Individuals are advised not to gain body mass during exercise, as it represents a risk factor for the development of hyponatremia [33]. A series of field studies [34,35,36,37,38] observing the ad libitum fluid intake of a total of 136 soldiers undergoing 15 to 40 km long route marches has been published. Of that number of soldiers, only three gained body mass during exercise, suggesting that gains in body mass may be a rare occurrence during walking/jogging exercises. However, relative to these studies, the incidence of cases in the current study is substantially more important. In the above studies, the walks were performed outdoors in groups, and individuals carried loads ranging from 17 to 55 kg. We speculate that the greater distractions generated by the surrounding environments, possibility for discussions between individuals or the difficulty of the task may have diverted participants’ attention further away from a more systematic planning of fluid intake than in the current study. Moreover, the longer duration of the 15–40 km marches (compared to this study) may have allowed more time for the adjustment of fluid intake and, hence, correction of overhydration. 

Heart rate, rectal temperature and perceived exertion and heat stress were either not statistically different or, from a practical standpoint, impacted distinctly among exercise sessions. This observation fits with the fact that ad libitum water intake volume, the percentage of sweat loss replaced by water intake and dehydration level were not significantly different among exercise sessions. Wilk et al. [17] also demonstrated that ad libitum drinking led to nonsignificant changes in heart rate and rectal temperature among six cycling sessions in young boys. Unfortunately, they neither assessed perceived exertion nor perceived heat stress. Our observations coupled with those of Wilk et al. [17] are important. In fact, they indicate to athletes, recreationally active individuals, and coaches that the dehydration-driven perturbations in heart rate, core body temperature and perceived exertion [39], all of which may be important modulator of exercise performance [4], are likely to be similar among relatively uniform exercise sessions when fluid is being consumed ad libitum. And this may have important implications in the planning of exercise, as alluded to in the introductory paragraph of this section.

The findings of the current study must be interpreted with the following limitations in mind. The results only apply to low-intensity walking/jogging exercises of 1 h. Higher intensity exercises of longer duration or sports requiring the driving of a machine at high speed (i.e., cycling) could reduce individuals’ focus and attention for bodily cues driving fluid requirements. In turn, this could result in less reproducible fluid intake volumes among similar exercise sessions. The participants were neither made aware that their water consumption was being monitored during exercise nor were they able to visualize the amount consumed at each gulp. However, participants had access to the elapsed time and, as such, it cannot be ruled out that they may have timed each intake of water according to a consciously or subconsciously determined schedule. Unfortunately, we did not monitor the elapsed time between each intake of water. Therefore, the reproducibility of water consumption among exercise sessions may have been, at least in part, confounded by this factor. Aside from the 1 h exercise sessions, participants were required to perform two other weekly exercise sessions during which they were asked to fully replace their sweat losses through water intake. It cannot be discounted that this drinking pattern may have influenced the drinking behavior of some participants during the 1 h exercise sessions. As shown in Table 2, urine specific gravity was significantly different among exercise sessions at arrival at the laboratory. Moreover, it amounted to 1.021 g·mL^−1^ prior to the fourth exercise session. Based on this observation, some may argue that individuals did not start each exercise session similarly or adequately hydrated. However, in healthy adults, dehydration or hypohydration is characterized by a urine specific gravity that exceeds 1.03 g·mL^−1^ [40]; participants were below this threshold prior to each exercise session. Moreover, thirst was low prior to each exercise session, and body mass fluctuations among exercise sessions were <1%. These latter two indices are suggestive of adequate hydration [40,41]. 

## 5. Conclusions

The current findings indicate that ad libitum water consumption results in reproducible fluid intake volumes among 1 h walking/jogging exercise sessions completed under identical conditions of temperature, humidity and exercise intensity. Moreover, associated changes in fluid balance status, heart rate, rectal temperature, perceived thirst, heat stress and exertion are expected to be relatively similar among exercises completed under identical scenarios and with ad libitum drinking. Nevertheless, our results highlight that ad libitum drinking may lead to water overconsumption in some individuals. Hence, as a precautionary measure, individuals completing novel exercise sessions while drinking ad libitum should be encouraged to check their body mass following exercise and adjust fluid intake during subsequent exercise sessions if need be. 

## Figures and Tables

**Table 1:** Physical characteristics of the participants.

Physical Characteristics	Mean ± SD
Age (years)	23 ± 5
Body mass (kg)	71.7 ± 12.7
Height (cm)	173.8 ± 8.5
Body mass index (kg·m^2^)	23.7 ± 3.7
Fat-free mass (kg)	57.9 ± 10.4
Relative maximal O_2_ consumption (mL·kg body mass·min^−1^)	52.7 ± 7.1
Resting heart rate (beats·min^−1^)	70 ± 13
Resting systolic blood pressure (mmHg)	119 ± 10
Resting diastolic blood pressure (mmHg)	73 ± 4

SD: standard deviation.

**Table 2:** Hydration-related variables upon arrival at the laboratory before each of the four exercise sessions.

Variable	Exercise Session 1	Exercise Session 2		Exercise Session 3		Exercise Session 4	*p*-Value
Urine-specific gravity (g·mL^−1^)	1.011 ± 0.007	1.016 ± 0.009		1.018 ± 0.008		1.021 ± 0.005	0.001
Body mass (kg)	72.1 ± 12.6	72.3 ± 12.7		72.3 ± 12.9		71.7 ± 12.8	0.18
Thirst (AU)	2.2 ± 0.8	2.3 ± 0.8		2.3 ± 0.8		2.7 ± 0.9	0.66
	Exercise Sessions 1 to 2		Exercise Sessions 1 to 3		Exercise Sessions 1 to 4		
Change in body mass (%)	0.35 ± 1.32		0.21 ± 1.55		−0.55 ± 1.45		0.046

AU: arbitrary unit. Data are the means ± SD.

**Table 3:** Balance between the total accumulated water intake volume and total accumulated sweat loss for each of the exercise sessions and over the four exercise sessions.

Participants	Exercise Session 1 (mL) *	Exercise Session 2 (mL) *	Exercise Session 3 (mL) *	Exercise Session 4 (mL) *	Total Accumulated Water Intake Volume (mL)—Total Accumulated Sweat Loss (mL) *
1	−650	−332	−303	−175	−1460
2	−1286	−1070	−1042	−1264	−4662
3	−580	−357	−72	+39	−970
4	−150	−50	+50	−200	−350
5	−500	−750	−150	0	−1400
6	−145	−466	−635	−245	−1491
7	+654	+414	+144	+312	+1524
8	−250	+200	+650	+400	+1000
9	−1150	−700	−250	−400	−2500
10	−800	−650	−500	−650	−2600
11	−700	−250	−200	−250	−1400
12	+1350	+450	−100	−400	+1300
Mean ± SD	−350 ± 738	−297 ± 476	−201 ± 419	−236 ± 439	−1084 ± 1783

* A negative value denotes an accumulated water intake volume < than the accumulated sweat loss. A positive value denotes an accumulated water intake volume > than the accumulated sweat loss. SD: standard deviation.

## Data Availability

Data will be made available from the corresponding author upon reasonable request.
